# The Role of Natural Antimicrobials in Reducing the Virulence of *Vibrio parahaemolyticus* TPD in Shrimp Gut and Hepatopancreas Primary Cells and in a Post-Larvae Challenge Trial

**DOI:** 10.3390/ijms26146557

**Published:** 2025-07-08

**Authors:** Lavinia Stef, Ioan Pet, Cosmin Alin Popescu, Gabi Dumitrescu, Liliana Petculescu Ciochina, Tiberiu Iancu, Iuliana Cretescu, Nicolae Corcionivoschi, Igori Balta

**Affiliations:** 1 Faculty of Bioengineering of Animal Resources, University of Life Sciences King Mihai I from Timisoara, 300645 Timisoara, Romania; laviniastef@usvt.ro (L.S.); ioanpet@usvt.ro (I.P.); gdumitrescu@animalsci-tm.ro (G.D.); lilianapetculescuciochina@animalsci-tm.ro (L.P.C.); 2 Faculty of Agriculture, University of Life Sciences King Mihai I from Timisoara, 300645 Timisoara, Romania; cosmin_popescu@usvt.ro; 3 Faculty of Management and Rural Development, University of Life Sciences King Mihai I from Timisoara, 300645 Timisoara, Romania; tiberiuiancu@usvt.ro; 4 Faculty of Medicine, Department of Functional Sciences, Victor Babes University of Medicine and Pharmacy, 2 Eftimie Murgu Square, 300041 Timisoara, Romania; iuliana.cretescu@umft.ro; 5 Bacteriology Branch, Veterinary Sciences Division, Agri-Food and Biosciences Institute, Belfast BT4 3SD, UK; 6 Academy of Romanian Scientists, Ilfov Street, No. 3, 050044 Bucharest, Romania

**Keywords:** *Vibrio parahaemolyticus (Vp*_TPD_), virulence, natural antimicrobials, translucent post-larvae disease (TPD), gut primary cells, hepatopancreas primary cells, oxidative stress, gene expression

## Abstract

Some *Vibrio parahaemolyticus* strains cause translucent post-larvae disease (*Vp*_TPD_), leading to significant economic losses in shrimp farming. We aimed to identify whether a mixture of natural antimicrobials, AuraAqua (Aq), can protect white-leg shrimp (*Penaeus vannamei*) against the lethal effects of *Vp*_TPD_ and to understand its biological mode of action. Herein, we demonstrate that Aq, an antimicrobial mixture composed of a blend of organic acids, citrus, and olive extracts, suppressed *Vp*_TPD_ virulence at sub-inhibitory concentrations and conferred robust protection to shrimp. The minimum inhibitory and bactericidal concentrations against the *Vp*_TPD_ isolate were at 0.05% and 0.2%, respectively. At 0.05–0.1%, Aq reduced bacterial growth and downregulated six major virulence genes (*vhvp-1*, *vhvp-2*, *vhvp-3*, *pir*A*_Vp_*, *pir*B*_Vp_*, *pir*AB*_Vp_)*, while leaving metabolic *ldh* expression unaltered. Parallel in vitro assays revealed diminished adhesion of *Vp*_TPD_ to primary shrimp gut and hepatopancreas epithelial cells and a ≈50% reduction in infection-induced extracellular H_2_O_2_, indicating an antioxidant effect. The treatment also triggered a time-dependent surge in extracellular alkaline phosphatase (ALP) activity, consistent with membrane permeabilization. In vivo, a challenge of post-larvae with 10^4^ CFU/mL *Vp*_TPD_ resulted in 91% mortality after 45 h; co-treatment with 0.1% and 0.2% Aq reduced mortality to ≈12% and ≈6%, respectively, while 1% Aq achieved ≈98% survival. The clinical protection test confirmed that 0.1% Aq preserved high survival across four pathogen inocula (10^1^–10^4^ CFU/mL). Conclusively, Aq destabilized the pathogen and therefore transcriptionally silenced multiple virulence determinants, translating into significant in-pond protection for controlling *Vp*_TPD_ for shrimp aquaculture.

## 1. Introduction

The translucent post-larvae disease (TPD) is a severe condition affecting shrimp, which is linked to *Vibrio* strains harboring the *Vibrio* highly virulent (*vhv*) gene [1]. Comparative genomic analysis of 16 *Vibrio* plasmids carrying the *vhv* gene revealed significant findings regarding their genetic diversity, transposon diversity, and associated secretion systems [1]. Isolated plasmids contained the Type IV Secretion Systems (T4SSs), specifically T4SS_typeT and the T4SS_typeF gene clusters. Notably, these plasmids, distinguished by their secretion system types, can coexist within single bacterial strains, underlying the pathogen’s potential for horizontal transfer of virulence traits. In this context, finding solutions to mitigate the negative impact of TPD is even more stringent since the amount of shrimp produced worldwide through farmed aquaculture has increased 10-fold, from less than 0.6 million tons in 1980 to over 5 million tons in 2022 and is estimated to increase to 7.28 million tons by 2025 [2]. From the different species of shrimp raised across the world, the two that are most popular are the white-leg shrimp (*Litopenaeus vannamei*) and the giant tiger shrimp (*Penaeus monodon*). Collectively, these species account for more than 90% of total farmed shrimp production. Notably, *P. vannamei* has become increasingly favored over *P. monodon* across multiple regions, driven primarily by several advantageous factors [2]. This includes the ability to breed many SPF (specific pathogen-free) broodstock, reduce dietary protein needs and related costs, cope well with different environmental and water-quality situations, be appreciated for their unique flavor, and supply a higher-quality diet [2].

The defining virulence factors of the TPD strain are two large exotoxins recently named *Vibrio* High Virulence Protein 1 and 2 (VHVP-1, VHVP-2), recently described by Liu and coworkers [3]. The two novel proteins were identified by isolating high-molecular-weight protein fractions from *Vp*_TPD_ that reproduced TPD disease in challenge experiments. For instance, *vhvp-2* appeared indispensable for virulence; therefore, Liu et al. created a ∆*vhvp-2* mutant that lost almost all lethality (causing <5% mortality, similar to non-pathogenic strains). Interestingly, complementation with *vhvp-2* restored full virulence, while a strain carrying *vhvp-1* alone was avirulent, suggesting *vhvp*-1 by itself is not toxic without its partner. From a molecular viewpoint, it implies a “two-component” toxin mechanism, where VHVP-1 (likely a TcA subunit) facilitates delivery or binding and VHVP-2 (TcB/C) executes the toxic effect. Structurally, VHVP-1 contains a receptor-binding and membrane translocation apparatus (including a neuraminidase-like domain), while VHVP-2 may act as the enzymatic toxin that disrupts host cells [3]. Apart from the toxin genes, the *Vp*_TPD_ genome retains many typical *V. parahaemolyticus* features that aid infection and environmental survival. Draft genome analysis (chromosomes I and II) of the TPD strain shows intact Type III Secretion Systems (T3SS1s and T3SS2s) on the chromosomes [4]. Another mechanism of *Vp*_TPD_ pathogenicity was also recently attributed to its potent Tc toxin complex, initially characterized in *Photorhabdus luminescens*, known for its sophisticated intracellular mode of action [5]. Structurally, this toxin complex is reported to comprise three distinct subunits—Tca, Tcb, and Tcc—each performing specialized roles in the virulence mechanism. In the literature, TcA was described as the initiator of the infection, which binds to the host cell membrane and enables subsequent membrane penetration, creating a channel for toxin translocation. Once established, a TcB-TcC complex containing a proteolytically activated toxic component is reported to be directly injected into the host cell cytoplasm, executing its lethal biochemical activity intracellularly. Additional investigations found that the toxin (i.e., TcA, TcB, and TcC) subunits are causative agents in *Vp*_TPD_-related mortality [4,6]. These Tc toxins mediate their pathogenic effects via the penetration of the host membranes, inducing disruptions of cellular function and potentially targeting cytoskeletal components (e.g., Myosin heavy chain, actin, and tropomyosin) as reported by LC-MS/MS analysis of infected shrimp fecal samples [7]. Overall, such toxins could contribute directly or indirectly to muscle tissue breakdown, leading to disruption of the digestive tract integrity.

In shrimp infected with *Vp*_TPD_, critical insights into the toxin’s pathogenic mechanisms were revealed by Huang et al., 2025 [5], which identified a dual pathogenic mechanism—direct cytoskeletal destabilization by Tc toxin and manipulation of host immune defences. Particularly, the presence of muscular cytoskeletal proteins, including Myosin heavy chain type 2, Fast-type skeletal muscle actin 15, Actin T2, and Tropomyosin, reflects a major disruption of muscle tissue inside the shrimp’s digestive system. The authors proposed that the observed cytoskeletal deterioration results either from direct proteolytic activity by Tc toxin or indirectly through toxin-induced cellular responses affecting these structural proteins [5].

Recent epidemiological data confirmed that multiple *Vibrio* isolates from TPD cases carried an identical plasmid-borne virulence factor, suggesting horizontal transfer of the toxin genes across strains and even species [3,8]. As a result, TPD may be caused by different *Vibrio* spp. after they acquire the main plasmid, making biosecurity issues more widespread; in practice, hatcheries now implement strict disinfection, early diagnosis, and exclusion protocols to curb *Vp*_TPD_, since traditional antibiotic use is forbidden for these reasons (resistance and export restrictions, respectively) [3]. Conventional control of *Vibrio* outbreaks in aquaculture has relied on antibiotics and strict hygiene, but antibiotic use is increasingly limited by regulatory and resistance issues [3]. As a result, researchers have explored diverse alternative strategies to prevent or mitigate *Vp*_TPD_ and associated infections. Some of the approaches to control and mitigate pathogens include natural antimicrobial compounds, bacteriophage therapy, and the use of beneficial microbes (probiotics) to outcompete or antagonize the pathogen. The individual antimicrobial ingredients are not usually used as separate supplements, as it has been suggested that blends of antimicrobial substances act better than as individual components [9]. The biological mechanisms of how a mixture of natural antimicrobials (AuraAqua) can prevent the onset of severe diseases were recently described in a fish infection model of *Lactococcus garvieae* [10]. The findings of this study describe the Aq direct antibacterial effect by reducing the expression of bacterial exopolysaccharide and haemolysis-related genes. In vitro, Aq reduced the pathogens’ adhesion to fish epithelial cells, inhibited the proinflammatory response, and prevented fish red blood cell lysis. These results highlighted the likely role of Aq as a non-antibiotic intervention to control fish lactococcosis, allowing better management of aquaculture disease. With this work, we have aimed to further describe its role in reducing *Vp*_TPD_ virulence, which will open new avenues of research, allowing us to better understand and design interventions to mitigate the devastating effects of *Vp*_TPD_ infections in shrimp. We also aimed to bring further evidence on its antioxidant role through hydrogen peroxide inhibition, a molecule involved in pathogen survival in the gastrointestinal tract. The overall aim of this study was to investigate, for the first time, the efficacy of a mixture of natural antimicrobials (AuraAqua) in preventing *Vibrio parahaemolyticus* TPD-like strain (*Vp*_TPD_) infection of shrimp gut and hepatopancreas primary cells. Moreover, we have also tested its effectiveness in vivo in a post-larvae challenge trial.

## 2. Results

### 2.1. Strain Identification and Genetic Characterization

**Figure 1:**
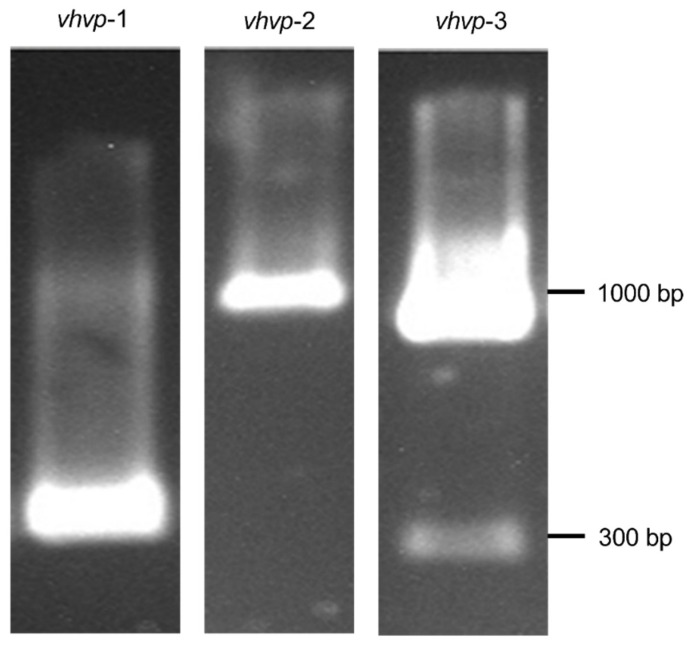
End point PCR results for the identification of *vhvp*-1, *vhvp*-2, and *vhvp*-3.

First, we aimed to prove that the strain used in this study encodes the virulence factors responsible for inducing the “translucent post-larvae disease” and causing high mortality rates in the post-larval stages of *Penaeus vannamei*. We first identified the presence of the *Vp*_TPD_ *vhvp*-1 gene, a key virulence factor of *Vp*_TPD_. The *vhvp*-2 gene was also identified, which in *Vp*_TPD_ is responsible for *V. parahaemolyticus’* lethal virulence in shrimp post-larvae. We have also detected the presence of *vhvp*-3, a potential key virulence gene of *Vp*_TPD_ with a lethal impact on post-larvae shrimp (Figure 1).

### 2.2. MIC/MBC and Growth Curves

**Figure 2:**
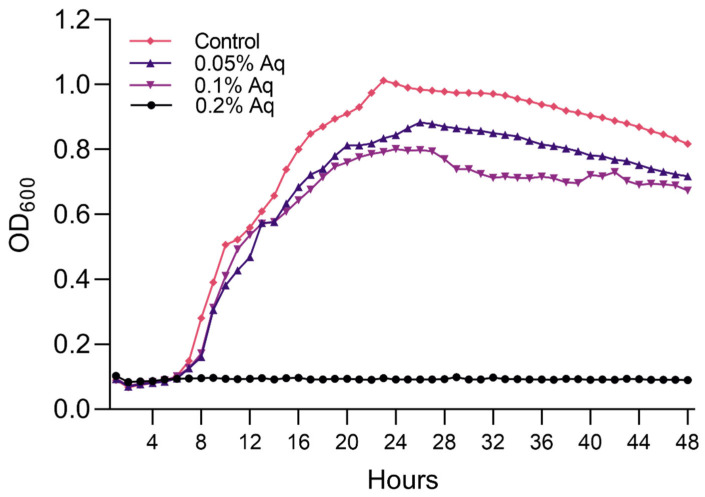
The impact of Aq on the *Vibrio parahaemolyticus* TPD strain growth. The MIC concentrations used are indicated on the graphs (0.05%, 0.1% and 0.2%). The experiments were performed in triplicate and on three separate occasions. To quantify the growth, the absorbance was measured at 600 nm every 4 h for 48 h. One-way ANOVA (*p* < 0.0001) and Dunnett’s tests were used to test for significance: Control vs. 0.2% Aq (*p* = 0.0001), Control vs. 0.1% Aq (*p* = 0.01), Control vs. 0.05% gave a *p*-value of 0.1.

The MIC and MBC cut-off points were established at 0.05% and 0.2%, respectively. To further identify the effect of the antimicrobial mixture on the bacterium’s virulence, we used the concentrations of 0.05% and 0.1% to avoid a lethal effect; however, these concentrations disrupted the growth of both strains (Figure 2). A significant *p*-value of <0.0001 resulted from the One-way ANOVA analysis. Dunnett’s multiple comparisons test identified a significant difference between the control group and the MBC value of 0.2% (*p* < 0.0001) and the MIC value of 0.1% (*p* = 0.01). No significance was identified when the control group was compared to the MIC value of 0.05% Aq (*p* = 0.1). These results show that these sublethal concentrations (0.1% and 0.05%) can be further used to assess the implications of virulence.

### 2.3. The Role of Aq in Preventing Vp_TPD_ Virulence and Reducing Oxidative Stress, in Vitro, in SGP and HP Cells

**Figure 3:**
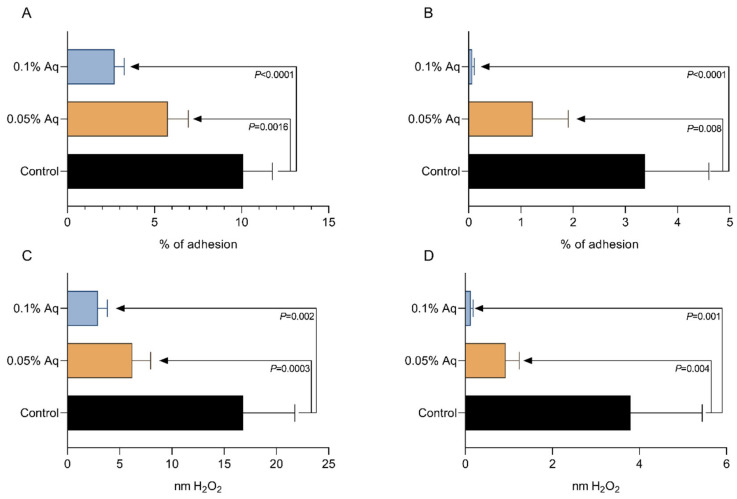
*Vp*_TPD_ total adhesion to SGP (**A**) and HP (**B**) cells in the presence of Aq. The results are expressed as percentages of the initial inoculum. The extracellular levels of H_2_O_2_ released by the infected SGP cells are presented in Figure (**C**) and the levels released by the HP cells in Figure (**D**). The significance levels are indicated on the graph. Significant differences were analyzed using the Student’s *t*-test with *p-*values indicated on the graph. One-way ANOVA (*p* < 0.0001) and Dunnett’s test for multiple comparisons both indicated significance. Error bars represent the standard deviation of means from three different experiments.

To correlate the negative effect on bacterial growth with a potential decrease in virulence, we performed in vitro infection assays using shrimp gut primary cells (SGPs) and hepatopancreas primary cells (HPs). The significance of the results was tested by using the Student’s *t*-test and the One-way ANOVA, followed by Dunnett’s test for multiple comparisons. The presence of Aq during the infection of SGP cells with the *Vp*_TPD_ strain significantly reduced the total adherence at both concentrations (Figure 3A). A similar pattern of reduced infection was observed when HP cells were infected (Figure 3B). The reduction was significant at 0.05% Aq (*p* = 0.008) and at 0.1% Aq (*p* < 0.0001). We have also investigated the impact of Aq in reducing oxidative stress in the infected SGP (Figure 3C) and HP (Figure 3D) cells by measuring the extracellular levels of H_2_O_2_. As shown in these two panels, a significant reduction in the H_2_O_2_ released was detected in both cases in the presence of 0.05% and 0.1% Aq. The *t*-test resulted in *p*-values that are indicated on the graphs (Figure 3). The One-way ANOVA analysis indicated that Aq has a significant protective effect against infection in both the SGP (*p* < 0.0001) and the HP cells (*p* = 0.0001) challenged with *Vp*_TPD_ (Figure 3B). Moreover, the significance of Aq in preventing in vitro infection was also confirmed by Dunnett’s test of multiple comparisons. The *p*-values obtained following Dunnett’s test analysis were 0.0003 (Control vs. 0.05% Aq) and <0.0001 (Control vs. 0.1% Aq) when SGP cells were infected. Similarly, when HP cells were infected, the *p*-values obtained following Dunnett’s analysis were 0.002 (Control vs. 0.05% Aq) and <0.0001 (Control vs. 0.1% Aq), respectively. The levels of H_2_O_2_ released by both the SGP (Figure 3C) and HP cells (Figure 3D) were also significantly decreased in the presence of Aq when analyzed by One-way ANOVA (*p* < 0.0001). Following multiple comparisons analysis by Dunnett’s test, the decrease was also significant (*p* = 0.0003 for Control vs. 0.05% Aq and <0.0001 for Control vs. 0.1% Aq) in infected SGP cells. Following similar analysis, in the infected HP cells, *p*-values of 0.001 for Control vs. 0.05% Aq and of 0.0001 for Control vs. 0.1% Aq were obtained. These results indicate that the reduced in vitro infection abilities of the *Vp*_TPD_ strain can be potentially reflected in vivo and accompanied by a reduced oxidative response.

### 2.4. Alkaline Phosphatase Activity

**Figure 4:**
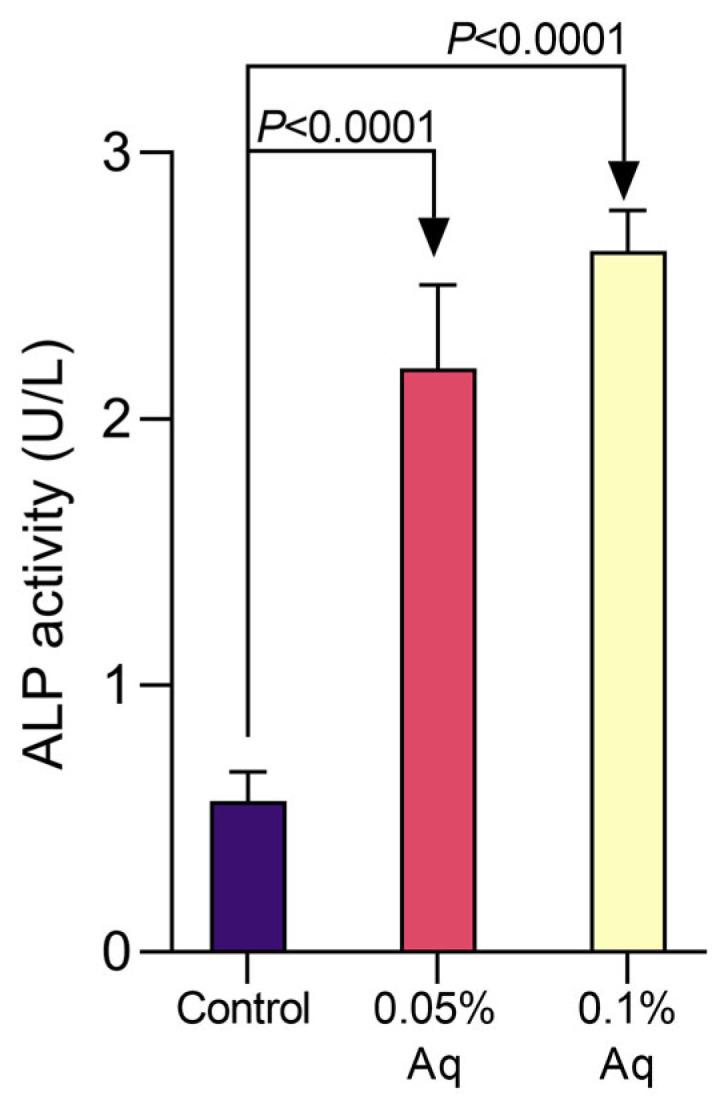
Changes in the antimicrobial activity of *V. parahaemolyticus* TPD strain and damage to its membrane permeability by extracellular alkaline phosphatase (ALP) in the bacterial supernatants following exposure to 0.05% and 0.1% Aq at 48 h. Significant differences were analyzed using the Student’s *t*-test with the *p-*values indicated on the graph. Error bars represent the standard deviation of means from three different experiments. A *p*-value of <0.0001 was also observed following analysis by One-way ANOVA and Dunnett’s multiple comparisons test.

The effect of Aq on ALP activity in the bacterial supernatant is shown in Figure 4. A small increase in ALP is observed in *Vp*_TPD_ cultures in the absence of Aq, as shown in Figure 4 (control). However, this increase was significantly accentuated in the presence of 0.05% Aq, with a significant increase after 48 h of growth by using the *t*-test analysis (*p* = 0.0001). This increasing trend in ALP activity was also detected in the presence of 0.1% Aq, with a significant increase at 48 h (*p* = 0.0005). The *p*-values resulting from the *t*-test analysis are indicated in Figure 4. In addition, a similar level of significance was detected by One-way ANOVA and Dunnett’s multiple comparisons test (*p* < 0.0001), indicating a significant difference between the control, in the absence of Aq, and the bacterial culture exposed to 0.05% and 0.1% Aq. These results clearly show that the antimicrobial mixture has time-dependent activity in bacterial cultures.

### 2.5. The Effect of Aq on Vp_TPD_ Virulence Gene Expression

**Figure 5:**
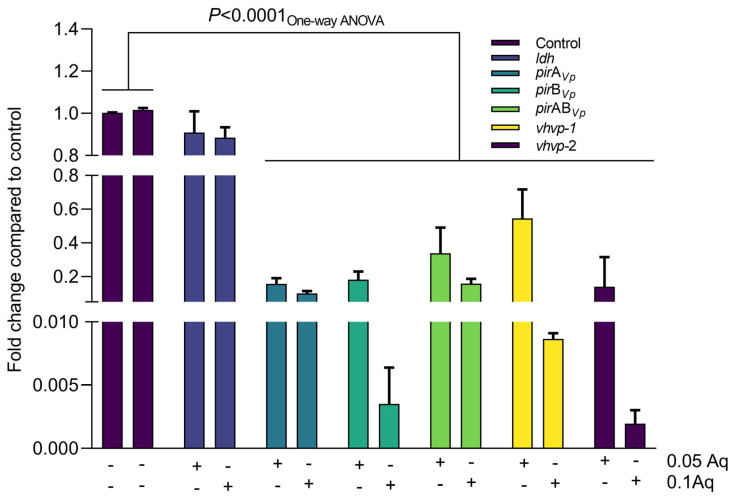
Effect of the antimicrobial mixture Aq on *Vp*_TPD_ on the expression of *vhvp*-1, *vhvp*-2, *vhvp*-3, *ldh*, *pir*A_Vp_, *pir*B_Vp_, and *pir*AB_Vp_ genes. Student’s *t*-test was used to account for significance, and *p*-values are represented on the graph. The significance observed with the *t*-test was also confirmed by Dunnett’s test analysis. Two-way ANOVA indicated a significant impact of Aq on the expression of all genes (*p* < 0.0001) except for the *ldh* gene, where the differences were not significant. Error bars represent the standard deviation of means from three different experiments performed in triplicate. The relative quantity of the mRNA was calculated using the ΔΔCt method. The 16S rRNA gene was used as an endogenous control since it was transcribed at equal rates in both the treated and untreated cells.

Next, we have investigated the impact of Aq on the expression of the *Vp*_TPD_ main virulence genes, including *vhvp*-1, *vhvp*-2, *vhvp*-3, *ldh*, *pir*A_Vp_, *pir*B_Vp_, and *pir*AB_Vp_ (Figure 5). The data presented in Figure 5 reflect the impact of Aq, at concentrations of 0.05% and 0.1%, at 48 h of growth. Our first observation was that the expression of the *ldh* gene was not affected by Aq at both concentrations. However, the expression of *vhvp*-1, *vhvp*-2, *vhvp*-3, *pir*A_Vp_, *pir*B_Vp_, and *pir*AB_Vp_ genes was significantly downregulated at both concentrations. The significance of Aq’s impact in bacterial gene regulation was also tested with Two-way ANOVA, followed by Dunnett’s test for multiple comparisons. The results were evaluated to determine the statistical significance of differences between the control and treated groups. The comparisons showed that in the case of the *ldh* gene (control vs. 0.05% Aq and control vs. 0.1% Aq), the test indicated no statistically significant differences with adjusted *p*-values of 0.9292 and 0.9237, respectively. These findings suggest that the treatments at 0.05% and 0.1% concentrations do not produce a significant effect compared to the control group for the measured parameter in the case of the *ldh* gene. For all the other genes investigated, through a similar comparison (control vs. 0.05% Aq and control vs. 0.1% Aq), the test indicated statistically significant differences in all cases (*p* < 0.0001). Few exceptions regarding the significance level were observed, in the case of the *pir*A*_Vp_* (*p* = 0.0001) and *vhvp*-2 (*p* = 0.0009) when the control was compared to 0.05% Aq. These results clearly suggest that at the subinhibitory concentrations of 0.05% and 0.1%, Aq can reduce the pathogenic abilities of *Vp*_TPD_.

### 2.6. VpTPD Challenge Test and the Clinical Protection Test

**Figure 6:**
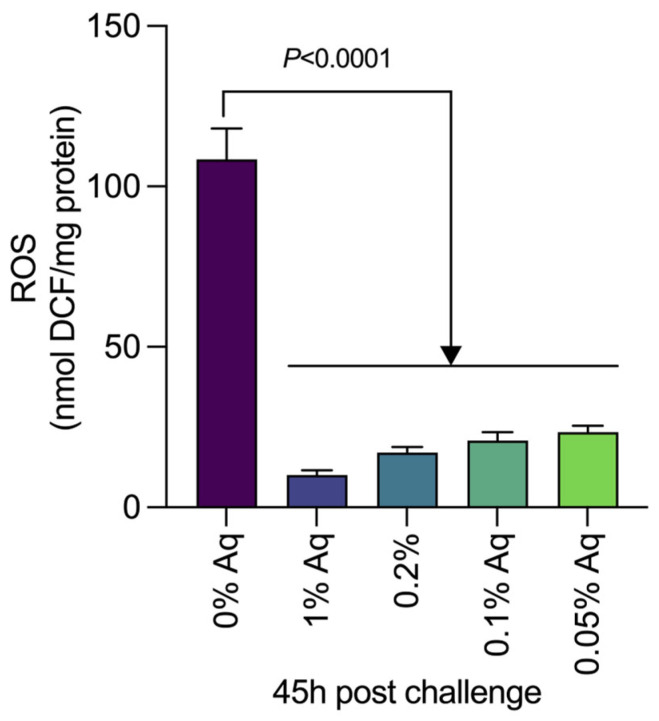
The levels of ROS detected in the hepatopancreas of challenged shrimp and after exposure to Aq. A *p*-value of <0.0001 was obtained using One-way ANOVA. Dunnett’s test indicated a *p*-value of <0.0001 between 0% Aq (Control) and all the other Aq concentrations individually. Error bars represent the standard deviation of means from three different experiments performed in triplicate.

To further estimate the beneficial effect of Aq, we have performed a challenge study by infecting *P. vannamei* shrimps with the *V. parahaemolyticus* TPD strain. Our results show that the decrease in mortality correlates with the MIC/MBC concentrations established (Table 1). The challenge study confirmed the MIC/MBC results through their impact on the bacterial growth, highlighting the efficiency of the antimicrobial mixture. The percentage of mortality decreased as the concentration of the antimicrobial mixture increased. These results clearly indicate that the antimicrobial mixture has the potential to protect the shrimp populations in vivo. Additionally, we have measured the levels of oxidative burst (ROS) in the hepatopancreas of the challenged shrimp. Data presented in Figure 6 clearly indicate that the presence of Aq significantly (*p* < 0.0001) reduces the levels of ROS in the infected tissue. The *p*-values obtained by using the *t*-test are indicated in Figure 6. The levels of significance following One-way ANOVA and Dunnett’s test for multiple comparisons analysis were similar to those resulting from the *t*-test analysis, as indicated in the figure legend.

**Figure 7:**
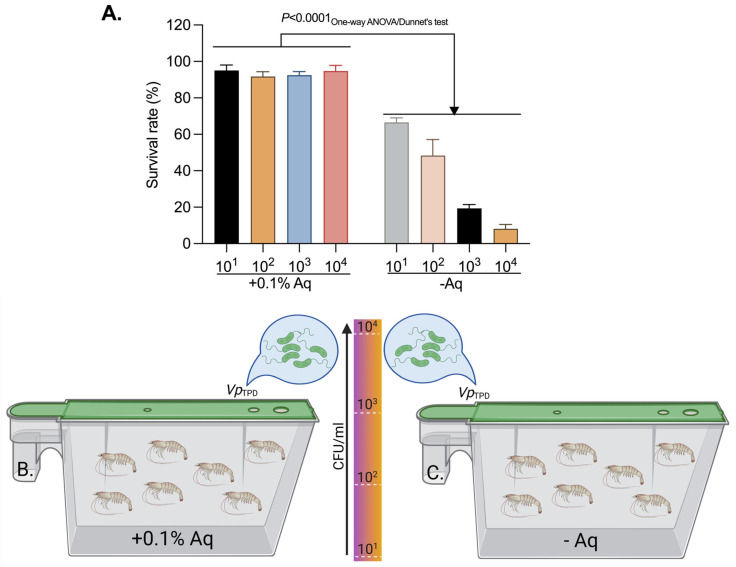
The survival rate of *P. vannamei* challenged with *Vp*_TPD_ at 10^1^, 10^2^, 10^3^, and 10^4^ CFU/mL (**A**) and schematic diagram of the clinical protection test without Aq (**B**) and in the presence of 0.1% Aq (**C**). Statistical significance was detected by using the Two-way ANOVA test to compare the groups in the presence or absence of Aq and Dunnett’s test (*p* < 0.0001) for multiple comparisons. The experimental diagram was designed using Biorender.com.

Next, we have evaluated the protective effect of Aq in shrimp challenged with different concentrations of *Vp*_TPD_. To investigate the protective effect of Aq on *P. vannamei* post-larvae challenged with 10^1^, 10^2^, 10^3^, and 10^4^ CFU *Vp*_TPD_/mL in the presence of 0.1% Aq (Figure 7B,C). This concentration was chosen due to the complexity of the experiment, taking into consideration that both 0.05% and 0.1% Aq showed similar efficacy. The control tank (Figure 7A) received no Aq. The results presented in Figure 7A clearly suggest that Aq has a protective effect at all bacterial concentrations investigated, suggesting a protective effect from the very early stage of infection. In contrast, in the absence of Aq, an increase in bacterial load led to a significant reduction in the shrimp survival rates (*p* < 0.0001). A similar significance (*p* < 0.0001) was detected when Dunnett’s test was used for multiple comparison to compare each bacterial load with the corresponding group in the control group in the presence of Aq.

## 3. Discussion

The shrimp farming sector has faced recurrent bacterial epidemics, notably due to *Vibrio* species. A decade ago, *V. parahaemolyticus* was identified as the etiological agent of Acute Hepatopancreatic Necrosis Disease (AHPND or “early mortality syndrome”) in shrimp, linked to plasmid-encoded PirA and PirB toxins [11,12]. In 2019–2020, a new highly virulent *V. parahaemolyticus* strain emerged in Asia, causing “Translucent Post-Larvae Disease” (TPD), also known as Highly lethal Vibrio disease (HLVD), bacterial-vitrified syndrome, or glass post larvae disease (GPD)—an outbreak distinct from AHPND [5]. TPD devastated shrimp hatcheries in coastal China, with 70–80% of nurseries collapsing during spring 2020 [3,7,13,14]. Morbidity is acute with the post-larval shrimp (PL4–PL7 stage) succumbed within ~3 days and with a cumulative mortality often reaching 100% [3]. Histopathology consistently shows acute necrosis and sloughing of hepatopancreatic tubule epithelium and midgut lining, which typically resembles AHPND lesions, but the speed and severity in TPD are greater [5]. The persistent recurrence implies that *Vp*_TPD_ may survive in environmental reservoirs or within sub-clinically infected populations, while analogous “early mortality” syndromes have been noted elsewhere (e.g., a “Zoea-2 syndrome” in India affecting larvae and “Las Bolitas syndrome” in Latin America affecting zoeal stages) [15]. TPD-affected larvae exhibit an empty digestive tract and pale colorless hepatopancreases, which become transparent (“glass post-larvae”) [13]. A *V. parahaemolyticus* isolate with a hemolysin gene (Vp-JS20200428004-2) was confirmed as the causative agent and is now referred to as the *Vp*_TPD_ strain [3,15,16]. This emergent pathogen rapidly became a leading cause of larval shrimp disease in Asia, outpacing prior threats in hatcheries. As of 2023, TPD remains a significant issue in shrimp farms across China. The alarming potency of TPD raises urgent concerns within China, emerging as the global leader of *Vibrio parahaemolyticus,* and about its possible transmission to neighboring countries, where shrimp farming practices may be similarly affected and have a severe economic impact [17]. The *Vp*_TPD_ pathogen demonstrates a remarkably high toxicity, approximately 1000 times greater than that of the *V. parahaemolyticus* strain known to cause AHPND [3]. In our study, we show that the antimicrobial mixture AuraAqua (Aq) can reduce the expression of the main virulence genes in *Vp*_TPD_-like strains (*vhvp*-1, *vhvp*-2, *vhvp*-3, *ldh*, *pir*A_Vp_, *pir*B_Vp_, and *pir*AB_Vp_). A recurring theme in the above strategies is that many aim at specific molecular targets of *V. parahaemolyticus* pathogenicity, which represents a shift from classic antibiotics (which non-specifically kill bacteria) to precision interference with virulence. For example, the *vhvp/Tc* toxin genes of *Vp*_TPD_ are an Achilles’ heel—without them, the bacterium is essentially harmless to shrimp [3]. While we cannot easily “knock out” these genes in the field, we can neutralize their products. Moreover, we show that Aq was able not only to reduce the ability of *Vp*_TPD_ to attach to primary shrimp gut and hepatopancreas cells but was also able to reduce the post-infection oxidative stress burst in these cells. This ability to prevent in vitro infection was also expressed in vivo in a post-larvae challenge test.

The beneficial effects of Aq were previously demonstrated against other shrimp and non-shrimp pathogens. Against gregarines, like *Nematopsis messor*, Aq can efficiently reduce the pathogen’s ability to colonize shrimp’s intestinal cells in vitro and in vivo and the oxidative-induced cellular damage repairs epithelial integrity and enhances gut immunity [18]. The antimicrobial mixture was also proven to act as a prebiotic and stimulate the growth of host probiotics, such as *F. prausnitzii*, increase the production of short-chain fatty acid (SCFA) butyrate, improve substrate digestion, and prevent *V. parahaemolyticus* invasion of shrimp gut primary cells [11]. Clearly, according to our current results, the antimicrobial mixture will also be able to act as a prophylactic intervention based on the data resulting from the clinical trial test. Moreover, further experiments are required to integrate the phenotypic and gene expression results to clearly identify the possible mechanism of action, comparing the efficacy and potential advantages of AuraAqua with other alternative treatments discussed in the literature and addressing practical questions through field test experimentation and how addressing the critical point of how this product could be practically applied in real shrimp aquaculture.

## 4. Materials and Methods

### 4.1. Bacterial Identification, Growth, and Antimicrobial Mixture

The *Vibrio parahaemolyticus* TPD strain (origin Vietnam) was kindly donated by Kim Orth from the Department of Molecular Biology, University of Texas, Southwestern Medical Center, Dallas, TX, USA. The strain was grown overnight at 37 °C in nutrient broth (Oxoid, Basingstoke, UK). As previously described, we have employed a PCR detection method to detect and confirm that the *Vp*_TPD_ strain [16] DNA was extracted from two strains of the *Vibrio parahaemolyticus* TPD strain using the PureLink Invitrogen extraction kit. The PCR was run on the Techne Thermocycler using MyTaq Red Mastermix in the following conditions: 95 °C/5 min, 95 °C/30 s, 58 °C/30 s, 72 °C/30 s, and 2 °C/10 min. PCR products were visualized by electrophoresis in 2% agarose gels. Primers are presented in Table 1. The natural antimicrobial mixture, AuraAqua (Aq), contains 5% maltodextrin, 1% sodium chloride, 42% citric acid, 18% sodium citrate, 10% silica, 12% malic acid, 9% citrus extract, and 3% olive extract (w/w). The raw materials were supplied by Bio-Science Nutrition Ireland. Experiments were carried out in triplicate.

### 4.2. Determination of Minimum Inhibitory Concentrations

The minimum inhibitory concentration (MIC) and minimum microbicidal concentration (MBC) were determined for the *Vibrio parahaemolyticus* TPD strain. The strain was grown overnight at 37 °C in nutrient broth (Oxoid, Basingstoke, UK). The resulting stationary phase cultures were diluted using a nutrient broth to give a suspension containing approximately 10^6^ CFU/mL. The Aq concentrations were prepared in the nutrient broth to give a range of concentrations from 4% down to 0.05%. One mL of each Aq solution was transferred into separate sterile plastic bijou bottles, and 1 mL of *V. parahaemolyticus* TPD strain was added to each bottle. A positive control containing 1 mL of nutrient broth and 1 mL of the 10^6^ CFU/mL suspension was prepared. The negative control was 2 mL of uninoculated nutrient broth plus salt. The mixtures were incubated aerobically at 37 °C for 24 h. This procedure was repeated on three separate occasions. The MIC value was determined as the lowest concentration of Aq that showed no bacterial growth. After 24 h, the bijou bottles were observed for bacterial growth. If the broth was clear, indicating no bacterial growth, then 100 µL was spread-plated onto nutrient agar using a sterile glass spreader. This was repeated for all concentrations of Aq that appeared clear in the bijou container. All plates were incubated aerobically at 37 °C for 24 h. This procedure was repeated on three separate occasions. The MBC value was determined as the lowest concentration of Aq that showed no bacterial growth on the plates.

### 4.3. Gene Expression

Similarly, the experiment was also performed to investigate the effect on *vhvp*-1, *vhvp*-2, *vhvp*-3, *ldh*, *pir*A*_Vp_*, *pir*B*_Vp_*, and *pir*AB*_Vp_* gene expression during growth and exposure to 0.05% and 0.1% Aq over a period of 48 h (Table 2). The RNA was reverse transcribed using the Transcriptor First Strand cDNA Synthesis Kit (Roche, Buckinghamshire, UK) according to the manufacturer’s protocols. The mRNA levels were determined by quantitative RT-PCR using the QuantiNovaSYBR^®^ Green PCR Kit (Qiagen, Manchester, UK) on a LightCycler^®^ 96 (Roche, Buckinghamshire, UK). A total of 5 μL of SYBR Green master mixture was used in each reaction, along with 0.5 μL of 10 μM primer mixture, 3 μL of molecular grade water, and 1 μL of DNA sample. The PCR conditions were 2 min at 95 °C, followed by 40 cycles of 95 °C for 5 s, 60 °C for 10 s, and a final extension at 72 °C for 5 min. A total of 5 μL of SYBR Green master mixture was used in each reaction along with 0.8 μL of 20 μM primer mixture, 7.4 μL of molecular grade water, and 1 μL of DNA sample. The relative quantity of the mRNA was calculated using the ΔΔCt method. The 16S rARN gene was used as an endogenous control since it was transcribed at equal rates in both the treated and untreated cells.

### 4.4. Growth Curves

Growth curves were established for the *V. parahaemolyticus* TPD strain. Broth cultures were prepared in a nutrient broth and incubated overnight at 37 °C. These were diluted using the nutrient broth to give a final concentration of approximately 10^6^ CFU/mL. The MIC values for the *V. parahaemolyticus* TPD strain were also determined and used to establish its impact on the bacterial growth profile. Two-fold dilutions of this solution were prepared in a 96-well plate, giving a range of AuraAqua from 0.05% to 0.2%. The final volume of AuraAqua solution in each well was 90 µL. A bacterial suspension (10 µL) was added to each well and thoroughly mixed. The 96-well plate was sealed with plastic film, and the optical density was measured at 600 nm at intervals of 4 h over a 48 h period at 37 °C using a FLUOstar Omega automatic plate reader (BGM Labtech, Aylesbury, UK). This procedure was repeated on three separate occasions.

### 4.5. In Vitro Infection Assay in a Shrimp Gut Primary Epithelial Cell Line (SGP) and in a Hepatopancreas Primary Epithelial Cell Line During Infection

To prepare the primary cells (SGPs), *P. vannamei* gut tissue samples were harvested. The surfaces of prawns were surface sterilized by swabbing with either 70% alcohol or 10 ppm active chlorine as bleach. Prawns were decapitated, and individual tissues were dissected from the prawn. SGP cells were prepared as follows. The gut was removed from the prawn and placed in a solution containing 4× penicillin 10,000 IU/mL, streptomycin 10,000 mcg/mL, and fungi-zone 25 mcg/mL and dissected into small pieces by crossed scalpel blades. The tissue fragments were washed twice with gentle centrifugation (150× *g* for 5 min). Five mL 0.25% trypsin at pH 7.4 at room temperature was added for 30–60 min and stirred on a magnetic stirrer, washed twice, and the cells were put into a 25 cm plastic culture flask with growth medium. Cultures were incubated at 28 °C. Primary cells in 24 plastic well plates (Analab, Lisburn, Northern Ireland, UK) with 0.1% DMSO (Thermo-Fischer, Gloucester, UK) media supplemented with 20% fetal bovine serum (FBS), 100 µg of penicillin, 8% shrimp head extract, 6% salt solution, 20 ng of epidermal growth factor (Sigma-Aldrich, Gillingham, UK), and 10 IUS/mL human recombinant interleukin 2 (Sigma-Aldrich, UK). To prepare the hepatopancreas primary cell line (HP), a modified protocol was used as previously described [21]. Briefly, the anaesthetized shrimp were sterilized with 75% ethanol and the hepatopancreas were removed aseptically and transferred to the Leibovitz L-15 medium (Thermo-Fisher, Gloucester, UK) supplemented with 10% fetal bovine serum, ciprofloxicin (100 mg/mL), penicillin (100 IU/mL), streptomycin (100 mg/mL), and nystatin (25 mg/mL). The removed tissue was homogenized in a glass homogenizer, followed by harvesting at 1000 rpm for 3 min in a centrifuge, with an additional collection at 7000 rpm for 10 min. The resulting pellet was added to L-15 medium and resuspended by aspiration using a disposable Pasteur pipette. Cells were seeded in 75 cm^2^ culture flasks at a concentration of 10^6^ cells and incubated at 28 °C. A confluent monolayer of cells was obtained after 72 h. Viability was measured by the trypan blue exclusion method conducted on both floating cells and attached cells. Cells excluding the dye were considered viable even if they were not proliferating. The pH during all the experimental infection studies was maintained at neutral values (pH 7–7.2). To avoid variations in pH, the natural antimicrobial mixture was pH-equilibrated before inclusion in the infection study. Cells were infected in the presence of 0.05% and 0.1% AuraAqua, and the infected monolayers were incubated for 3 h followed by washing three times with the tissue culture media. Cells were infected with 10^4^ CFU/mL *V. parahaemolyticus* TPD strain. After infection, the infection media was removed, and the infected monolayers were washed three times with the tissue culture media. The infected cells were then incubated with tissue culture media containing gentamicin (100 µg/mL) to expose the internalized bacteria or to total lysis without gentamicin inclusion, but, instead, 0.1% Triton X was included to reveal the total bacterial adhesion.

### 4.6. Challenge Tests (Counting Living Larvae) and the Clinical Protection Test

The *V. parahaemolyticus* TPD-positive strain was tested for its pathogenicity by a challenge test using healthy *Penaeus vannamei* post larvae, following a procedure previously described [21]. Twenty-five shrimp post larvae per replicate were plated in sterile petri dishes and exposed to infection with 10^4^ CFU/mL bacteria for 10 min. The antimicrobial mixture was applied at the time of infection in concentrations of 0.05%, 0.1%, 0.2%, and 1% in 500 mL flasks. Survival was determined by counting the larvae at 45 h after infection. A positive and a negative control (±antimicrobial mixture or ±larvae) were also included in the challenge at 0% of the antimicrobial mixture. The experiment was performed in triplicate. For the clinical protection test, the *Vibrio parahaemolyticus* TPD strain was grown overnight at 37 °C in nutrient broth (Oxoid, Basingstoke, UK). Bacteria were then centrifuged at 6000 rpm for 10 min and diluted to concentrations of 10^1^, 10^2^, 10^3^, and 10^4^ CFU/mL. *Penaeus vannamei* post larvae were infected for 48 h in the presence or absence of Aq. For this experiment, only the concentration of 0.1% was selected, given the complexity of the experiment, and to reduce the use of biological materials. The levels of ROS in the hepatopancreas of challenged shrimp were measured as previously described [22].

### 4.7. Alkaline Phosphatase Activity (ALP)

This experiment was performed as previously described [23]. Briefly, the *V*. *parahaemolyticus* strain was cultured as described above and then centrifuged at 5000 rpm for 15 min, followed by resuspension in 1 M PBS to approximately 10^8^ CFU/mL and exposed to 0.05% and 0.1% Aq for 48 h. After incubation, the samples were centrifuged at 5000 rpm for 10 min at 4 °C to collect the supernatant. Alkaline phosphatase (AKP) activity was measured using the Alkaline Phosphatase Kit (ab83369, Abcam, Cambridge, UK). The absorbance of the supernatant was read at 520 nm using the microplate reader.

### 4.8. Extracellular Hydrogen Peroxide (H_2_O_2_) Measurements in Infected SGP and HP Cells

The amount of H_2_O_2_ released by the infected SGP cells was measured as previously described [24]. Briefly, an H_2_O_2_ Amplex^®^ UltraRed/HRP (Thermo Fischer, Gloucester, UK) kit was used according to the manufacturer’s instructions. The culture media (50 mL) was mixed with the Amplex^®^ UltraRed/HRP (Thermo Fischer Scientific, UK) reagent and with the horseradish peroxidase, resulting in a red fluorescent oxidation product. Fluorescence was determined at 530 nm excitation and 590 nm emission using a fluorescence microplate reader (FLUOstar Omega, BMG Labtech, Belfast, UK). The concentrations of H_2_O_2_ were calculated using standard curves. All experiments were performed in triplicate.

### 4.9. Statistical Analysis

Statistical analyses were performed using GraphPad software, version 11. Data were represented as mean ± SD. *p*-values < 0.05 were considered statistically significant following estimations using the Student’s *t*-test. One-way ANOVA, Two-way ANOVA, and Dunnett’s tests were used for grouped and multiple comparisons.

## 5. Conclusions

Herein, we have demonstrated that an antimicrobial mixture (Aq) administered at sub-inhibitory concentrations (0.05% and 0.1%) effectively attenuates the pathogenicity of *Vp*_TPD_, the causative agent of “translucent post-larvae disease” in *P. vannamei*. Aq suppressed bacterial growth, curtailed adhesion to shrimp gut and hepatopancreas primary cells, and markedly lowered extracellular H_2_O_2_ release—an early indicator of host oxidative stress—thereby interrupting the initial stages of infection. From a molecular perspective, we observed a down-regulation of the essential virulence determinants *vhvp-1*, *vhvp-2*, *vhvp-3*, *pir*A*_Vp_*, *pir*B*_Vp_*, and *pir*AB*_Vp_*, while *ldh* expression remained unaffected, indicating that Aq selectively targets virulence markers rather than basal metabolism. Simultaneously, ALP activity implied compromised membrane integrity, which proposes a possible mechanistic linkage between Aq exposure and loss of bacterial fitness. In vivo challenge assays corroborated these findings, showing shrimp mortality decline from 91% in untreated controls to 12% at 0.1% Aq and to 2% at 1% Aq, with protection evident even when larvae were challenged with up to 10^4^ CFU/mL of the pathogen. In conclusion, our data suggest a dual mechanism whereby Aq (1) disrupts the pathogen, triggering membrane-associated stress responses, and (2) silences key toxin genes, eventually alleviating host oxidative injury and enhancing shrimp survival.

## Figures and Tables

**Table 1:** Mortality of *Penaeus vannamei* after 4 h of challenge after infection with the *V. parahaemolyticus* TPD strain.

Aq Concentration (%)	Mortality (%)
0.05	34.7 ± 3.7
0.1	11.5 ± 2.2
0.2	5.9 ± 7.1
1	2.1 ± 3.2
0	91.4 ± 6.7

**Table 2:** List of primers used in this study.

Gene Name	Primer Sequence	Reference
*vhvp*-1	F acgactgacccggtacgcatgtayatgmgngaratgggnacngt	[19]
	R atagaaataaccagacgtaagttngcytcnaccatytcyttyt,	
*vhvp*-2	F ggagtattggtgggctgaaa	
	R ggtaggcatggaccgtaaag	
*vhvp*-3	F agagtttgatcmtggctcag	
	R ggytaccttgttacgactt	
*ldh*	F aaagcggattatgcagaagcactg	[19]
	R gctactttctagcattttctctgc	
*pir*A*_Vp_*	F tgactattctcacgattggactg	[19]
	R cacgactagcgccattgtta	
*pir*B*_Vp_*	F tgatgaagtgatgggtgctc	
	R tgtaagcgccgtttaactca	
*pir*AB*_Vp_*	F gcaccgtaaattttcaggtt	[20]
	R cgttgcaatctaagacatag	

## Data Availability

All the data is included in the study.

## References

[1] Zhang Y., Tan P., Liang X., Zhang Q., Yang M. (2025). Vibrio plasmids harboring vhv gene associated with shrimp translucent post-larvae disease: Coexistence of two types of T4SS and multiple transposons. J. Invertebr. Pathol..

[2] Wikumpriya G.C., Prabhatha M.W.S., Lee J., Kim C.-H. (2023). Epigenetic Modulations for Prevention of Infectious Diseases in Shrimp Aquaculture. Genes.

[3] Liu S., Wang W., Jia T., Xin L., Xu T.t., Wang C., Xie G., Luo K., Li J., Kong J. (2023). Vibrio parahaemolyticus becomes lethal to post-larvae shrimp via acquiring novel virulence factors. Microbiol. Spectr..

[4] Rosilan N.F., Jamali M.A.M., Sufira S.A., Waiho K., Fazhan H., Ismail N., Sung Y.Y., Mohamed-Hussein Z.-A., Hamid A.A.A., Afiqah-Aleng N. (2024). Molecular docking and dynamics simulation studies uncover the host-pathogen protein-protein interactions in Penaeus vannamei and Vibrio parahaemolyticus. PLoS ONE.

[5] Huang Z., Liao Y., Du J., Yang Z., Li F., Ruan L., Shi H. (2025). Transcriptomic insights into the resistance mechanism of Penaeus vannamei against highly lethal Vibrio parahaemolyticus. Sci. Rep..

[6] Yang F., You Y., Lai Q., Xu L., Li F. (2023). Vibrio parahaemolyticus becomes highly virulent by producing Tc toxins. Aquaculture.

[7] Lin H.-Y., Lin H.-T., Chiang Y.-R., Lu Y.-Y., Pan J., Chen M.-H., Chuang J.-C., Kuo W.-C., Lin H.-J., Lin J.H.-Y. (2024). Herbmedotcin™ as an Alternative Antimicrobial Agent for Hatcheries Rearing Pacific White Shrimp (Litopenaeus Vannamei). SSRN.

[8] Jia T., Liu S., Yu X., Xu T., Xia J., Zhao W., Wang W., Kong J., Zhang Q. (2024). Prevalence investigation of translucent post-larvae disease (TPD) in China. Aquaculture.

[9] Toschi A., Rossi B., Tugnoli B., Piva A., Grilli E. (2020). Nature-Identical Compounds and Organic Acids Ameliorate and Prevent the Damages Induced by an Inflammatory Challenge in Caco-2 Cell Culture. Molecules.

[10] Balta I., Simiz F.D., Stef D., Pet I., Dumitrescu G., Iancu T., Cretescu I., Corcionivoschi N., Stef L. (2025). A New Strategy to Prevent Emerging Lactococcus garvieae Infections by Using Organic Acids as Antimicrobials In Vitro and Ex Vivo. Int. J. Mol. Sci..

[11] Butucel E., Balta I., McCleery D., Marcu A., Stef D., Pet I., Callaway T., Stef L., Corcionivoschi N. (2022). The Prebiotic Effect of an Organic Acid Mixture on Faecalibacterium prausnitzii Metabolism and Its Anti-Pathogenic Role against Vibrio parahaemolyticus in Shrimp. Biology.

[12] Lin S.-J., Huang J.-Y., Le P.-T., Lee C.-T., Chang C.-C., Yang Y.-Y., Su E.C.-Y., Lo C.-F., Wang H.-C. (2022). Expression of the AHPND Toxins PirAvp and PirBvp Is Regulated by Components of the Vibrio parahaemolyticus Quorum Sensing (QS) System. Int. J. Mol. Sci..

[13] Intriago P., Medina A., Espinoza J., Enriquez X., Arteaga K., Aranguren L.F., Shinn A.P., Romero X. (2023). Acute mortality of Penaeus vannamei larvae in farm hatcheries associated with the presence of Vibrio sp. carrying the VpPirAB toxin genes. Aquac. Int..

[14] Yu P., Shan H., Cheng Y., Ma J., Wang K., Li H. (2022). Translucent disease outbreak in Penaeus vannamei post-larva accompanies the imbalance of pond water and shrimp gut microbiota homeostasis. Aquac. Rep..

[15] Intriago P., Montiel B., Valarezo M., Romero X., Arteaga K., Cercado N., Burgos M., Shinn A.P., Montenegro A., Medina A. (2024). Las Bolitas Syndrome in Penaeus vannamei Hatcheries in Latin America. Microorganisms.

[16] Jia T., Xu T., Xia J., Liu S., Li W., Xu R., Kong J., Zhang Q. (2023). Clinical protective effects of polyhexamethylene biguanide hydrochloride (PHMB) against Vibrio parahaemolyticus causing translucent post-larvae disease (VpTPD) in Penaeus vannamei. J. Invertebr. Pathol..

[17] Zakaria N.H., Abd Rahim N.D.E., Rosilan N.F., Sung Y.Y., Waiho K., Harun S., Zainal Abidin R.A., Afiqah-Aleng N. (2025). Global landscape of Vibrio parahaemolyticus research: A bibliometric analysis. World J. Microbiol. Biotechnol..

[18] Balta I., Stef L., Butucel E., Gradisteanu Pircalabioru G., Venig A., Ward P., Deshaies M., Pet I., Stef D., Koyun O.Y. (2022). The Antioxidant Effect of Natural Antimicrobials in Shrimp Primary Intestinal Cells Infected with Nematopsis messor. Antioxidants.

[19] Vicente A., Taengphu S., Hung A.L., Mora C.M., Dong H.T., Senapin S. (2020). Detection of Vibrio campbellii and V. parahaemolyticus carrying full-length pirABVp but only V. campbellii produces PirVp toxins. Aquaculture.

[20] Phiwsaiya K., Charoensapsri W., Taengphu S., Dong H.T., Sangsuriya P., Nguyen G.T.T., Pham H.Q., Amparyup P., Sritunyalucksana K., Taengchaiyaphum S. (2017). A Natural Vibrio parahaemolyticus Δ*pirA^Vp^ pirB^Vp^*^+^ Mutant Kills Shrimp but Produces neither Pi*^rVp^* Toxins nor Acute Hepatopancreatic Necrosis Disease Lesions. Appl. Environ. Microbiol..

[21] Uma A., Prabhakar T., Koteeswaran A., Ravikumar G. (2002). Establishment of primary cell culture from hepatopancreas of Penaeus monodon for the study of white spot syndrome virus (WSSV). Asian Fish. Sci..

[22] Wei K., Yang J. (2015). Oxidative damage of hepatopancreas induced by pollution depresses humoral immunity response in the freshwater crayfish Procambarus clarkii. Fish. Shellfish. Immunol..

[23] Ding M., Xu Z., Ding Y., Song X., Ding C. (2025). High-Efficiency Electrochemiluminescence Biosensor with Antifouling and Antibacterial Functions for Sensitive and Accurate Analysis of Chloramphenicol in Seawater. Anal. Chem..

[24] Corcionivoschi N., Alvarez L.A., Sharp T.H., Strengert M., Alemka A., Mantell J. (2012). Mucosal reactive oxygen species decrease virulence by disrupting Campylobacter jejuni phosphotyrosine signaling. Cell Host Microbe.

